# Intrinsic Noise Analyzer: A Software Package for the Exploration of Stochastic Biochemical Kinetics Using the System Size Expansion

**DOI:** 10.1371/journal.pone.0038518

**Published:** 2012-06-12

**Authors:** Philipp Thomas, Hannes Matuschek, Ramon Grima

**Affiliations:** 1 School of Biological Sciences, University of Edinburgh, Edinburgh, United Kingdom; 2 SynthSys Edinburgh, University of Edinburgh, Edinburgh, United Kingdom; 3 Department of Physics, Humboldt University of Berlin, Berlin, Germany; 4 Institute of Physics and Astronomy, University of Potsdam, Potsdam, Germany; Virginia Tech, United States of America

## Abstract

The accepted stochastic descriptions of biochemical dynamics under well-mixed conditions are given by the Chemical Master Equation and the Stochastic Simulation Algorithm, which are equivalent. The latter is a Monte-Carlo method, which, despite enjoying broad availability in a large number of existing software packages, is computationally expensive due to the huge amounts of ensemble averaging required for obtaining accurate statistical information. The former is a set of coupled differential-difference equations for the probability of the system being in any one of the possible mesoscopic states; these equations are typically computationally intractable because of the inherently large state space. Here we introduce the software package intrinsic Noise Analyzer (iNA), which allows for systematic analysis of stochastic biochemical kinetics by means of van Kampen’s system size expansion of the Chemical Master Equation. iNA is platform independent and supports the popular SBML format natively. The present implementation is the first to adopt a complementary approach that combines state-of-the-art analysis tools using the computer algebra system Ginac with traditional methods of stochastic simulation. iNA integrates two approximation methods based on the system size expansion, the Linear Noise Approximation and effective mesoscopic rate equations, which to-date have not been available to non-expert users, into an easy-to-use graphical user interface. In particular, the present methods allow for quick approximate analysis of time-dependent mean concentrations, variances, covariances and correlations coefficients, which typically outperforms stochastic simulations. These analytical tools are complemented by automated multi-core stochastic simulations with direct statistical evaluation and visualization. We showcase iNA’s performance by using it to explore the stochastic properties of cooperative and non-cooperative enzyme kinetics and a gene network associated with circadian rhythms. The software iNA is freely available as executable binaries for Linux, MacOSX and Microsoft Windows, as well as the full source code under an open source license.

## Introduction

Chemical kinetics is by its very nature stochastic. This stochasticity has several origins, chief among them being that spontaneous processes are responsible for the conformational changes which occur in unimolecular reactions while the process of bringing two molecules together to participate in a bimolecular reaction is Brownian motion [1]. This randomness is averaged out and hence non-apparent when the reactions under study involve a large number of molecules. This is the case of reactions occurring in test-tubes or in even larger systems. However inside cells, conditions are such that many species exist in low copy numbers [2]. The importance of stochasticity is particularly obvious in genetic regulatory networks since there are one or two copies of most genes per cell [3]. It is thus clear that stochastic modeling of intracellular networks is necessary to understand the complex biochemical processes underpinning a cell’s response to both internal and external perturbations.

Current software implementations offer a broad range of stochastic modeling methods. Available packages can be divided into particle based descriptions and population based descriptions. Particle based methods adopt a microscopic approach that describes the movement of each individual reactant (non-solvent) molecule in space and time by means of Brownian dynamics. Popular software packages include the Greens Function Reaction-Diffusion algorithm [4], Smoldyn [5] and MCell [6]. Population based methods adopt a mesoscopic approach that retains the discreteness of reactants but does not need to simulate individual particle trajectory explicitly. This methodology, used by packages such as Smartcell [7] and MesoRD [8], is based on the reaction diffusion master equation [9], [10]. The basic idea is to divide the reaction volume into smaller subvolumes, with reactions proceeding in each subvolume and molecules entering adjacent subvolumes by diffusion. Next one applies the well-mixed assumption to each subvolume (but not to the whole system) which implies that we can ignore the positions and velocities of individual molecules inside each subvolume. The state of the system is then described by the number of molecules of each species in each subvolume, a description which is considerably reduced compared to that offered by particle based methods. This methodology relies on the knowledge of length scales over which the system is said to be spatially homogeneous [2].

A further reduced population description can be achieved by specifying to the situation in which the concentrations of interacting molecules are approximately spatially homogeneous over the entire reaction volume. Reaction kinetics is governed by two timescales: (i) the diffusion timescale, i.e., the time it takes for two molecules to meet each other and (ii) the reaction timescale, i.e., the time it takes for two molecules to react when they are in close proximity to each other. Concentration homogeneity over the entire compartment in which reactions occur, ensues when the reaction timescale is much larger than the diffusion timescale [2]. The large majority of available software packages, deterministic or stochastic, model this situation. Under such well-mixed conditions the Stochastic Simulation Algorithm (SSA) provides an accurate mesoscopic description of stochastic chemical dynamics. The SSA is a Monte Carlo technique by which one can simulate exact sample paths of the stochastic dynamics. The latter has been rigorously derived from microscopic physics by Gillespie for dilute well-mixed gases and solutions [11], [12]. Over the past two decades, the popularization of the algorithm has led to its broad availability in many software packages (see Table 1). However in many situations of practical interest, the application of the SSA is computationally expensive mainly due to the two reasons: (i) whenever the fluctuations are large, e.g., the case of low copy numbers of molecules, a considerably large amount of ensemble averaging of the stochastic trajectories is needed to obtain statistically accurate results. (ii) the SSA simulates each reaction event explicitly which becomes computationally expensive whenever the copy number of at least one molecular species is large [1].

**Table 1:** Current software approaches for stochastic modeling.

Package	REs	Stochastic Simulation				CME				GUI	SBML	Ref
			Mean	Var	PDF		Mean	Var	PDF			
BioNetS	✓	✓	✓	✓	✓					✓	✓	[72]
Cain	✓	✓_P_	✓	✓	✓					✓	✓	[73], [74]
CellMC		✓_P_									✓	[75]
Copasi	✓	✓			✓					✓	✓	[18]
Dizzy	✓	✓	✓							✓	✓	[76]
SimBiology	✓	✓_P_	✓	✓	✓					^(2)^	✓	[77]
StochKit		✓_P_	✓	✓	✓						^(4)^	[78]
StochPy	^(5)^	✓	✓	✓	✓					^(3)^	✓	[79]
CMEpy						✓FSP	✓	✓	✓	^(3)^		[14]
MomentClosure	✓					✓MA	✓	✓		^(1)^	✓	[15]
StochDynTools	✓					✓MA	✓	✓		^(2)^		[16]
iNA	✓	✓_P_	✓	✓		✓^SSE^_P_	✓	✓		✓	✓	

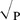
 implementation uses multi-core parallelism, ^(1)^ Maple, ^(2)^ Matlab, ^(3)^ matplotlib, ^(4)^ using converter, ^(5)^ if used as plugin for PySCeS [80], 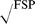 method based on the Finite-State Projection algorithm, 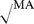 method based on moment approximation, 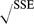 method based on system size expansion.

Existing software packages are divided in groups presenting implementations based on stochastic simulation or such based on the Chemical Master Equation. The software iNA combines both by using the system size expansion which has not been available in a software package yet. In particular, we also list whether the package allows for evaluation of mean concentrations, variances (Var) and the probability density function (PDF).

The chemical master equation (CME) is a mathematically equivalent and hence complementary description to the SSA [9]. The CME is a system of linear ordinary differential equations with an unbounded or a typically very large finite state space given by all combinations of copy numbers of the reactant molecules. Hence the advantage of the CME over the SSA is that it does not require any ensemble averaging and is not based on time-consuming simulation of individual reactions. However the CME does not lend itself easily to numerical or analytical computation, the reason being the large dimensionality of its state space. Hence to-date, software packages exploiting the utility of the CME have been scarce (see Table 1). Direct numerical integration of the CME is possible through the finite state projection method [13] which is implemented in the python package CmePy [14]. However, the state space grows exponentially with the number of species and hence these methods have limited applicability in biologically relevant situations. A different type of approach involves the calculation of the moments of the probability distribution solution of the CME by approximate means. Generally there exists an infinite hierarchy of coupled moment equations for reaction networks with bimolecular interactions. In order to make progress, a common method involves the truncation of the hierarchy by means of a moment-closure scheme. The software MomentClosure [15] implements the normal moment-closure approximation for mass action networks by setting all cumulants higher than a desired order to zero. A variety of alternative closures schemes are implemented in the package StochDynTools [16]. The advantage of these approaches is that they generally present quick ways to investigate the effects of noise without the need for averaging over many realizations of the stochastic process. However, these methods are based on ad hoc assumptions for the choice of the closure scheme and hence their accuracy and range of validity is often unknown.

In this article we introduce the software package, intrinsic Noise Analyzer (iNA), which enables a complementary approach using van Kampen’s system size expansion (SSE) of the CME together with traditional approaches of deterministic and stochastic simulation of chemical kinetics. The present implementation features the approximate computation of a selected number of moments of the probability distribution function solution of the CME by use of the Linear Noise Approximation (LNA) as well as the effective mesoscopic rate equations (EMREs). The advantage of these methods is that they are not based on ad-hoc assumptions like moment-closure approximations but rather they are based on the SSE which is a systematic expansion of the CME in powers of the inverse volume of the compartment in which reactions occur. The LNA provides the lowest order approximation to the second moments of the probability distribution (the variance and covariance of fluctuations) while the EMREs provide the first-order correction to the concentrations predicted by the deterministic rate equations (REs). iNA is the first software package to bridge the gap between deterministic simulation of chemical kinetics using rate equations, stochastic simulation of kinetics by the SSA and systematic analytic approximations of the CME. The novel combination of these complementary approaches makes iNA a valuable tool for the study of intrinsic noise in biological systems that has not yet been available to researchers and non-expert users in standalone software. The article is organized as follows. First we describe iNA’s input specifications, the implemented methods and the features of the GUI. We then present the use of iNA to explore the dynamics of three models commonly encountered in biochemical kinetics: non-cooperative enzyme kinetics, cooperative enzyme kinetics and a gene network associated with circadian rhythms. Finally we discuss the design and implementation of the SBML parser and the methods, along with optimizations that increase the performance of the analysis. We complete the presentation by a derivation of the SSE-based methods at the heart of iNA.

The software is available as executable binaries for Linux (Fedora, Ubuntu, OpenSuse), MacOSX (10.5, 10.6, 10.7) and Microsoft Windows (7, XP) from http://code.google.com/p/intrinsic-noise-analyzer, as well as the full source code under the open source GPL2 license. The SBML files used in this article are available under the same URL.

## Results

### Input Format Specification

The general formulation of biochemical kinetics considers a number N of distinct chemical species confined in a mesoscopic volume of size 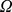 under well-mixed conditions. Species interact via *R* chemical reactions of the type
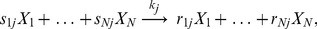
(1)


where *j* is the reaction index running from 1 to *R*, *X*_i_ denotes chemical species *i*, *k_j_* is the reaction rate of the *j^th^* reaction and *s_ij_* and *r_ij_* are the stoichiometric coefficients. We associate with each reaction a propensity function 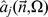 such that the probability for the *j^th^* reaction to occur in the time interval 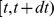 is given by 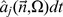. The vector 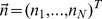 denotes a mesoscopic state where *n_i_* is the number of molecules of the *i^th^* species. Note that our general formulation does not require all reactions to be necessarily elementary, i.e., unimolecular and bimolecular chemical reactions, but can also describe effective reactions. If the *j^th^* reaction is elementary then its reaction rate *k_j_* is a constant while if it is non-elementary the reaction rate is a function of the instantaneous concentrations, i.e., the elements of the vector 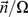.

This description of the biochemical reaction networks in terms of reactants, products, the associated stoichiometries and kinetic laws are part of the Systems Biology Markup Language (SBML) [17] which has become a standard representation of such networks. iNA natively supports SBML compatible with level 2 version 4 which makes the software versatile to work with models exchanged from other applications as Copasi [18], CellDesigner [19], SBML editor [20] or shorthand SMBL [21] as well as with some of the many models that are freely available in public databases [22]. The basic components of SBML parsed by iNA are definitions of units, compartments, species and reactions. iNA reads all SBML files that describe reaction networks as defined by the reaction scheme (1) along with the associated propensities. The former is obtained from SBML’s reactant and product stoichiometry definitions while the latter is parsed from the “KineticLaw” construct. Species can be specified in terms of both amount (*mol*, molecule numbers and derived units) and concentration (*molar*, number concentrations and derived units).

The validation of models considered suitable for stochastic analysis requires the software to make several restrictions on SBML model definitions. Currently, events and explicit time dependent rate parameters are not supported. Also, reactions defined by the SBML specific reversible attribute cannot be validated for stochastic models due to ambiguities in the associated “KineticLaw”. We refer users to the software package Copasi which allows for convenient conversion between SBML’s reversible and irreversible reaction definitions. For consistency, the definition of a species must conform all species to be defined non-constant and free of algebraic constraints. The implementation of iNA assumes that such constraints arise naturally from the stoichiometry of the reaction network. Furthermore it is required for all parameters to be evaluated before runtime of simulations. Therefore the software does not allow parameters to be defined by ODEs or assignment rules. The software package gives the appropriate error messages in cases where one of the above specifications is not met.

### Stochastic Simulation

Over the past two decades the SSA has enjoyed widespread popularity mainly because of the ease by which one can simulate stochastic reaction networks [23], [24]. Given that the system is in state 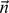 at time *t*, Gillespie proved using the laws of probability [25] that the probability per unit time for the *j^th^* reaction to occur at time 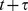 is
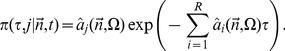
(2)


The SSA generates a stochastic trajectory of the kinetics by sampling a reaction index *j* and a reaction time 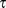 according to Eq. (2), followed by an update of the population size 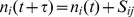 for every species *i*. Note that the net change of the molecule number of species *i* by reaction *j* is given by the stoichiometric matrix 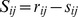. Despite its popularity, stochastic simulation has two major shortcomings. Firstly, simulations have to be carried out a significantly large number of times because of the considerable amount of independent realizations needed to obtain accurate statistical averages. Secondly, simulations can become quite slow when the population number of any molecular species is large [1]. Stochastic simulation is a basic component of the software iNA with support for simultaneous simulation of independent realizations using shared memory parallelism of the OpenMP standard [26]. The software features two implementations of the SSA via the direct and the optimized direct method [25], [27]. The output data is presented in terms of mean concentrations, variances and correlations as a function of time which allow for direct statistical interpretation.

### The Chemical Master Equation and the System Size Expansion

An equivalent formulation for the stochastic reaction network described by Eq. (1) is the CME which can be derived from combinatorial arguments [9], [10], [28] and or from microphysics [11], [12]. The CME gives the time-evolution equation for the probability 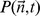 that the system is in a particular mesoscopic state 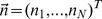,
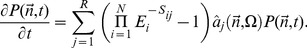
(3)


Here we have introduced the step operator 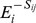 which is defined by its action on a general function of molecular populations as 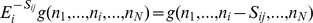 [9]. The CME is equivalent to a set of coupled differential-difference equations for each possible mesoscopic state, i.e., each combination of reactant molecule numbers. Typically, the large number of such states makes the CME intractable for numerical and analytical computation. The software package iNA uses an alternative approach based on van Kampen’s SSE of the CME which is applicable whenever the dynamics of the reaction network is monostable [9]. In brief, the method constitutes a large volume expansion whose successive terms can be used to approximate the moments of the probability density function to any desired accuracy. Thereby it is implicit that whenever the reaction volume is large (or equivalently the molecular populations are large at constant concentration) the average concentrations can be approximated by the macroscopic REs,
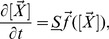
(4)


which are exactly the same as those used in deterministic models of biochemical kinetics. Note that 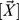 is the vector of macroscopic concentrations and 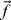 is the macroscopic rate function vector, see Methods section. Note that matrices are underlined throughout the article.

The leading order term of the SSE is given by the LNA which has been the key tool in analytical studies of noise [9], [29], [30]. The merit of the method is that it provides a simple means of calculating the fluctuations about the concentration solution of the REs. In particular one is typically interested in the covariance of the time-dependent concentrations
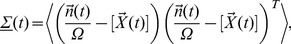
(5)where the angled brackets denote the statistical average. Within the LNA the elements of the covariance matrix are determined by the time-dependent equation



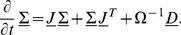
(6)Note that the matrix 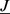 is the Jacobian which gives the extent by which small perturbations of the REs, Eq. (4), decay. The matrix 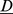 is the diffusion matrix which quantifies the size of the perturbation due to intrinsic noise. Both matrices can be constructed from the stoichiometric coefficients and the macroscopic rate function vector 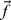. The diagonal elements of 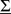 are the variances and hence determine the standard deviation of concentration fluctuations by
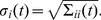
(7)


The off-diagonal elements are the covariances which determine Pearson’s correlation coefficients between the concentration fluctuations of species *X_i_* and *X_j_*.
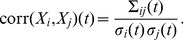
(8)


Considering higher terms of the expansion one can obtain corrections to the REs which stems from a coupling of the mean concentrations to the higher order moments of the concentration fluctuations. These corrections have been calculated for networks composed of elementary reactions by Grima [31]. The new time-evolution equations which are obtained from this analysis are called effective mesoscopic rate equations (EMREs) and they are here extended for general reaction networks composed of elementary and non-elementary reaction steps (see *Methods* section). The EMREs are given by
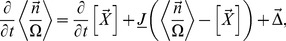
(9)


where 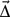 is a vector which describes the coupling of the mean concentrations to the variance and covariance of fluctuations in the concentrations. It depends on the macroscopic concentrations and the covariance matrix which can be obtained by the solution of Eq. (4) and (6), respectively. We refer the interested reader to the section *Methods* for the definition of the vector 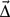 together with an explicit derivation of the prescribed methods. EMREs have been shown to accurately describe the mean concentration over a wide range of copy numbers [31]–[35]. Generally the predictions of the REs and the EMREs agree only for reaction networks of unimolecular reactions. For more general reaction networks such as those involving bimolecular or non-elementary reactions, EMREs provide finite-copy number corrections to the REs. It is to be kept in mind that EMRE’s provide meaningful results if and only if the predicted mean concentrations for all species are positive; in practice this means that the EMRE is valid for reaction volumes above a certain breakdown volume which is system specific (see [35] for further discussion).

The present software allows the computation of the SSE methods either in time dependent conditions or at steady state. The former is obtained by numerical integration of the set of coupled ordinary differential equations, Eqs. (4), (6) and (9). The latter uses the same set of equations with the time derivative set to zero and reduces them to a set of simultaneous algebraic equations which typically can be solved with less computational effort and higher numerical accuracy. For the first time, all of the the above methods are made available to a broad audience by the software package iNA.

### Features of the GUI

The software iNA aims at ease of use of analytical approximations that facilitate the exploration of stochastic effects in biochemical reaction networks. We have therefore focused on a minimal GUI composed of a model tree with table and plot views. Analyses tasks can be easily accessed through wizards which guide the user through configuration. The GUI is divided into a menu bar and a main window. After a model has been loaded using the menu bar it will be available in the list of “Open models” on the left hand side. The latter list is hierarchically organized as follows

**Figure 1:**
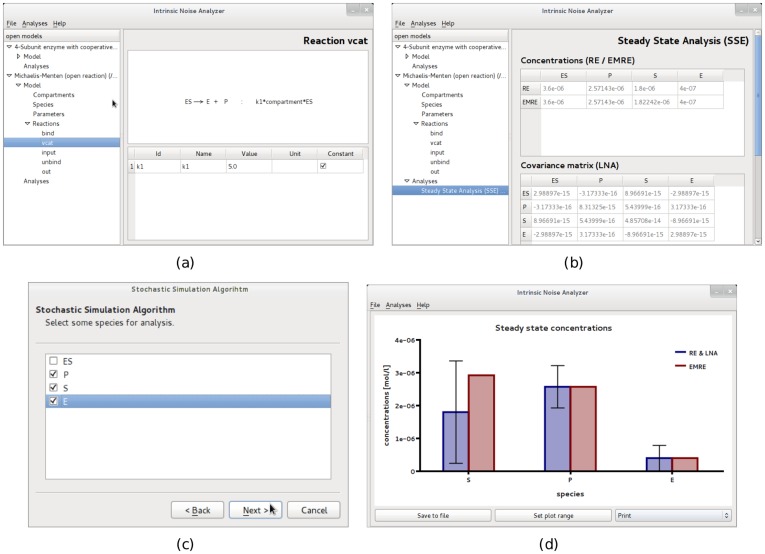
GUI of the software iNA. (a) *Model views* gives information on reactions, rate constants, propensities and species. (b) *Table views* provide the analysis results in an easy-to-read format. (c) iNA’s wizards allow for user friendly configuration of analyses. (d) *Plot views* visualize results in neat format. Note that SSE in (b) stands for system size expansion.
- *Analyses*, where the results of the Linear Noise Approximation, EMRE or SSA analyses can be accessed; see Fig. 1b.

- *Model*, where the basic components of the model can be accessed. These include compartments, species, parameters and reactions; see Fig. 1a.

The *Model* section contains a basic SBML reader for viewing the parameters that define the biological model and making sure that the SBML file has been parsed correctly. The *Analyses* section can be filled with items selected from the option *Analyses* in the menu bar. Currently there are three wizards available.

- a *Steady State Analysis (SSE)* wizard which customizes the computation of LNA, RE and EMRE in steady state conditions,
- a *Time Course Analysis (SSE)* wizard which customizes the computation of the LNA, RE and EMRE in time-dependent conditions,
- a *Stochastic Simulation Algorithm (SSA)* wizard guiding the initialization of stochastic simulations.

All wizards allow the user to select a subset of species to be analyzed, see Fig. 1c. The output of each analysis is simply a data table which can be saved to a text file or visualized by the predefined plot widgets.

The *Steady State Analysis* is the most basic analysis provided by the program. It yields mean concentrations and the covariance matrix according to the LNA. Note that to the LNA level of approximation, the mean concentrations are the same as those obtained from solving the REs. The roots of the REs are computed by iNA using the Newton-Raphson method with line search [36] and therefore it is required to specify precision and maximum number of iterations of the algorithm. iNA also outputs the mean concentration predictions according to EMREs [31]. These account for stochastic effects and hence are generally expected to be much closer to the true concentration prediction of the CME. Thereby one can obtain a quick estimate of the effect of noise on the reaction network. iNA offers convenient visualization of the outputs using the *Plot* option shown in Fig. 1d. This bar plot features two separate columns for the concentrations calculated by the REs and the EMREs of the individual species. The former is complemented with an error bar which indicates the standard deviation of the concentration fluctuations calculated using the LNA. The *Time Course Analysis* presents a wizard which is as simple to use as conventional integrators for deterministic REs by which one specifies the final time of integration, and the maximum relative and absolute errors. Therefore it is clear that all results obtained by the SSE should be checked for consistency with numerical integration carried out using smaller error estimates. The time course consists of theoretical estimates according to the deterministic REs and the corresponding fluctuations around it which have been estimated by the LNA at any point in time. At the same time the estimation of the mean concentrations using EMREs is shown and can be compared to its deterministic counterpart. The results can be accessed by a table view presenting the numerical data of the analysis or by selected plot views showing the mean concentrations and fluctuations computed by the REs and LNA, the correlation coefficients obtained from the LNA as a function of time or a comparison between the concentration predictions of EMREs and the REs.

In order to validate the results, the *Stochastic Simulation* wizard offers the choice between two different implementations of the SSA (see section Design and Implementation) and allows the user to adjust the number of independent realizations that are used to calculate the statistical averages. This enables direct comparison of the simulation results with those obtained from the analysis using the SSE methods. All outputs are exportable to text files.

### Applications

We showcase the utility of the present software by analyzing three models of biochemical kinetics: the Michaelis-Menten reaction [37], a multi-subunit enzyme with cooperative kinetics [38], [39] and a gene network with negative feedback that has been proposed for circadian rhythms in Drosophila and Neurospora [40]. The SBML files that have been used in this section are listed in Table 2.

**Table 2:** SBML model definition files that have been used in this article.

F1	enzymekinetics1.xml	Enzyme with Michaelis-Menten kinetics, 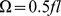 .
F2	enzymekinetics2.xml	Enzyme with Michaelis-Menten kinetics, 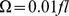
F3	coopkinetics1.xml	Multi-subunit enzyme with cooperative kinetics, 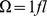
F4	coopkinetics2.xml	Multi-subunit enzyme with cooperative kinetics, 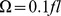
F5	coremodel1.xml	Circadian clock model, 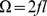 , weak negative feedback
F6	coremodel2.xml	Circadian clock model, 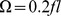 , weak negative feedback
F7	coremodel3.xml	Circadian clock model, 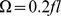 , strong negative feedback

All files and the software iNA are available from the URL http://code.google.com/p/intrinsic-noise-analyzer.

### Michaelis-Menten Reaction with Substrate Input

The Michaelis-Menten reaction is a well studied example of biochemical kinetics. Over the past decade, stochastic models of the reaction have received considerable attention by means of analytical and stochastic simulation methods [23], [24], [32], [41]. We here consider an embedded reaction mechanism that also accounts for flux conditions naturally found in living cells which comprises substrate input and product consumption reaction steps.
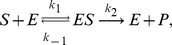


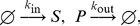
(10)


where *S* denotes the substrate species, *E* the free enzyme species, *ES* is the enzyme-substrate complex and *P* the product species. The *k*’s refer to the associated rate constants. In what follows, we consider the same reaction occurring in two compartments characterized by two different length scales: the cellular scale which is the scale of large organelles such as mitochrondria (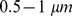) [42] and the scale of small sub-cellular compartments such as lipid rafts (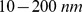) [43]. Two SBML files F1 and F2 (see Table 2) have been provided, one for each length scale. The rate constants are the same for both files and are shown in Table 3. These can also be conveniently accessed by the *Model view* of iNA, see Fig. 1a. Note that these rate constants are obtained from an experimental study of the enzyme Malate dehydrogenase [44].

**Table 3:** Rate constants for Michaelis-Menten kinetics with substrate input (SBML files F1 and F2).

*k*_in_	1.8×10^−5^ *M/s*	*k*_out_	7*s* ^−1^
*k*_1_	5×10^7^ (*Ms*)^−1^	*k*_−1_	5*s* ^−1^
*k*_2_	5*s* ^−1^		

#### Michaelis-Menten kinetics on the cellular scale

We here consider the reaction scheme (10) to take place in a compartment of volume 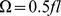 (femtoliters) which corresponds roughly to the size of a bacterium or a large organelle [45]. We now analyze the steady-state stochastic properties of the reaction by means of the LNA and the EMRE.

**Figure 2:**
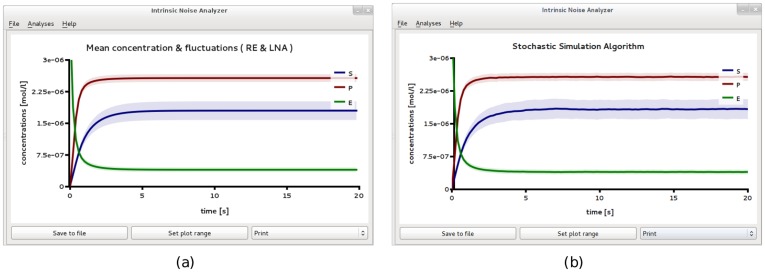
Michaelis-Menten kinetics in a large compartment of volume 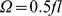. Plots showing the results of *Linear Noise Approximation* (a) and *Stochastic simulation* using an ensemble of independent 1,000 realizations (b). The two are in excellent agreement. Both figures have been obtained from iNA’s analysis of the SBML file F1.

The SBML file F1 specifies an initial condition of about 1200 enzyme molecules in non-complex form, which corresponds to a total enzyme concentration of 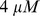 (micromolar); the substrate and product concentrations are initially zero. The model definition can be opened in iNA to perform the *Steady State Analysis*. The output generated is a table view which is shown in Fig. 1b that can also be exported to a text file as well as visualized by a plot (see Fig. 2a). We obtained values for the substrate and product concentrations to be 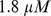 and 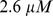 according to the REs. The table view gives further information on the intrinsic fluctuations in terms of the covariance matrix in steady state. We have computed the coefficient of variation (CV), a non-dimensional quantity which measures the inverse signal-to-noise ratio. The CV of species *X* is defined as 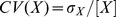, where 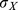 is the standard deviation defined by Eq. (7). We obtain 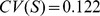 and 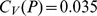. Note that the EMRE concentrations obtained from the *Steady State Analysis* of iNA are very close to those predicted by the REs. This suggests that the LNA predictions of CV are accurate.

We verified the accuracy of the LNA predictions by obtaining 1000 independent realizations using the *Stochastic Simulation* wizard; the result is shown in Fig. 2b. The figure shows that the concentrations have reached steady state after about 5*s*. We then exported the data to a file and averaged over the output for 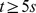. The resulting mean concentrations are given by 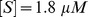 and 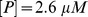 with mean variances of 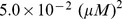 and 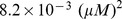, respectively. These values correspond to CV of about 0.124 and 0.035 which are in excellent agreement with the predictions of the LNA computed by iNA.

#### Michaelis-Menten kinetics in a small intracellular compartment

**Figure 3:**
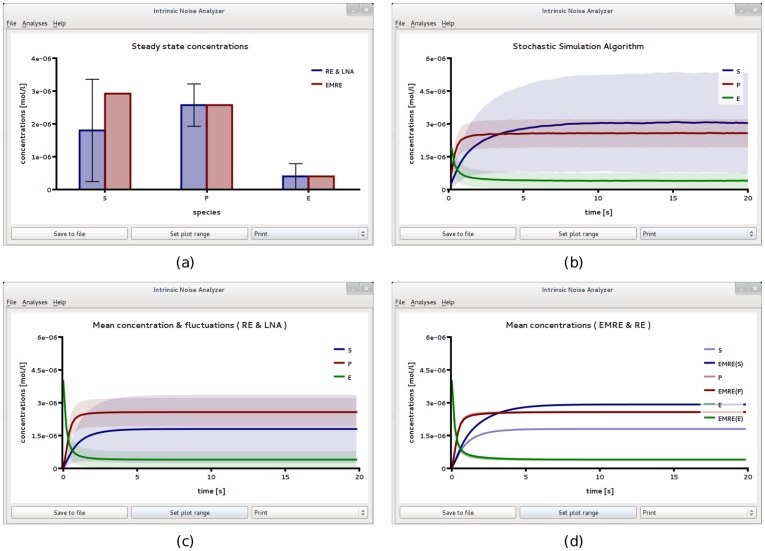
Michaelis-Menten kinetics in a small compartment of volume 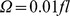. (a) Plot of iNA’s *Steady State Analysis* shows amplified EMRE substrate concentrations in comparison with those predicted by the REs. This conclusion is supported by stochastic simulations (b) using an ensemble size of 10,000 realizations which are in excellent agreement with steady state and time course predictions shown in (a) and (d), respectively. (c) For comparison we show the result of the LNA time course which fails to accurately predict both the mean substrate concentrations and the variance of fluctuations about them. The figures have been obtained from iNA’s analysis of the SBML file F2.

Next we study the same reaction in a compartment of reduced volume 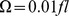 which roughly corresponds to a spherical volume of diameter 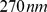. The enzyme concentrations correspond to a total copy number of only 24 molecules. In such a small compartment, the average substrate concentrations can be greatly enhanced by intrinsic noise. The *Steady State Analysis* performed by iNA is summarized in Fig. 3(a). The substrate concentration is 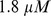 according to the RE and 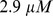 according to the EMRE. This implies a noise-induced enhancement of the concentration by about 60%. Note for all other concentrations – those of enzyme, complex and product – the predictions of the RE and EMRE theory agree exactly and thus are independent of the volume of the compartment. These phenomena have been found earlier by Grima [32] using the SSE and are well reproduced by the software iNA.

We verify these predictions by stochastic simulation; the result is shown in Fig. 3b. We can now compare this result to the time course obtained from the REs and the LNA, see Fig. 3c. A comparison of the latter two subfigures reveals that the approach to steady state as obtained from SSA simulations is significantly slower than the one predicted by the macroscopic REs. Note also that the variance of the substrate concentration of the SSA is considerably larger than that predicted by the LNA. However we see that the time course of the stochastic simulation in Fig. 3b is well reproduced by the EMRE, see Fig. 3d. We exported the data from stochastic simulation to a text file and performed a time average over concentrations for which 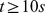. The results are 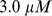 and 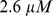 for substrate and product concentrations, respectively, which are in excellent agreement with the predictions of the EMREs. We have also estimated the coefficient of variation by stochastic simulations to be 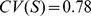. Note that in this case the LNA predicts a value of 0.86 which overestimates the size of intrinsic noise by about 10%.

Comparing Fig. 3b and 3c, shows that the relative magnitudes of the concentrations of substrate and product are reversed when noise is taken into account. In contrast, a comparison of Figs. 2a and 2b, shows that the relative magnitudes of the concentrations of substrate and product remain the same even when noise is taken into account. This indicates that a noise-induced concentration inversion effect occurs below some critical compartment volume, a phenomenon which has recently been described by Ramaswamy et al. for the trimerization reaction with bursty input and for a two gene circuit with negative feedback [35]. In the section *Methods*, we use EMREs to calculate the theoretical value for the critical volume at which the concentrations of substrate and product become equal. Above the critical volume, the product concentration is larger than that of the substrate and hence the REs are qualitatively correct. Below the critical volume, the substrate concentration is larger than that of the product and REs are then qualitatively incorrect. The critical volume is calculated to be 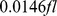 for the enzyme Malate dehydrogenase considered here. Note that the compartment volume used in this example (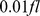) is smaller than the critical volume whilst the volume used in the previous example (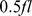) is much larger. This explains why the concentration inversion effect is only observed in the current example.

### Cooperative Enzyme Kinetics

Apart from Michaelis-Menten kinetics, it is often found that many enzymes exhibit positive cooperativity in substrate binding. Such behavior is a kinetic signature of enzymes with multiple interdependent binding sites. As a consequence, experimental ligand binding curves show a sigmoidal dependence on the abundance of specific substrates [37], [46]. Tyson [38] used REs to study the deterministic cooperative kinetics of a two subunit enzyme catalyzed system (an enzyme with two binding sites). Here we extend the model to involve four subunits and study its stochastic properties using iNA. The reaction scheme reads



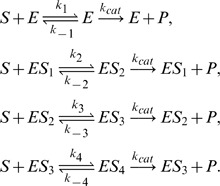
(11)


We enforce the condition 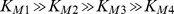 where 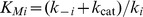 is the Michaelis-Menten constant of the *i^th^* enzyme reaction. This condition ensures that the binding of a substrate molecule to the enzyme molecule with *n* sites already occupied by other substrate molecules occurs quicker than substrate binding to an enzyme molecule with *n*−1 sites occupied; hence the phenomenon of positive cooperativity. In order to account for flux conditions that are found naturally in living cells, we also include substrate input and product removal reactions
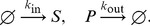
(12)


We have written two SBML model files, F3 and F4, that describe the chemical reactions with an initial total enzyme concentration of 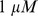 inside two different compartment volumes (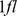 and 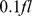). The rate constants are the same for both compartments and can be found in Table 4. The choice of rate constants leads to Michaelis-Menten constants which are in the physiological range [47] and which guarantee positive cooperativity: 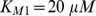, 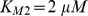, 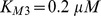 and 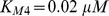.

**Table 4:** Rate constants for model of cooperative enzyme kinetics with substrate input (SBML files F3 and F4).

*k*_in_	9 *µM/s*	*k*_out_	10*s* ^−1^
*k*_1_	1(*µMs*)^−1^	*k*_−1_	10*s* ^−1^
*k*_2_	10(*µMs*)^−1^	*k*_−2_	10*s* ^−1^
*k*_3_	1×10^2^(*µMs*)^−1^	*k*_−3_	10*s* ^−1^
*k_4_*	1×10^3^(*µMs*)^−1^	*k*_−4_	10*s* ^−1^
*k_cat_*	10*s* ^−1^		

#### Cooperative enzyme kinetics at the cellular scale

**Figure 4:**
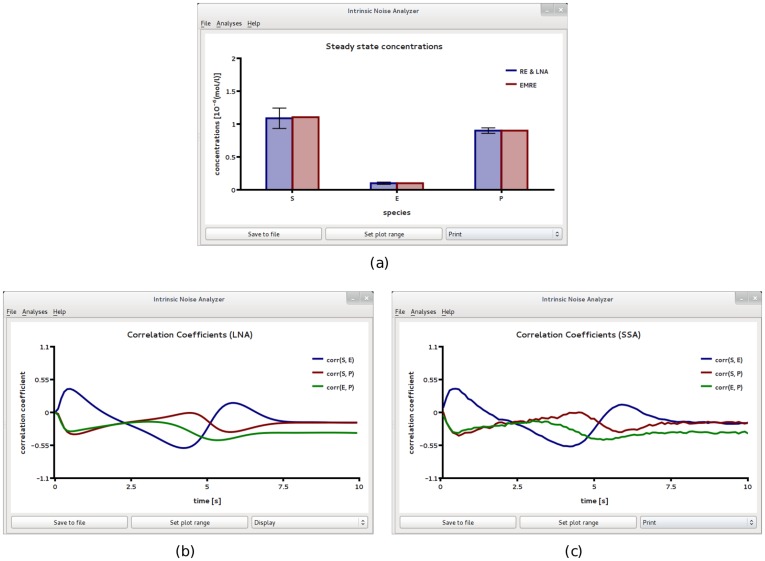
Cooperative enzyme kinetics in a large compartment of volume 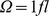. (a) Plot of the *Steady State Analysis* which shows good agreement of RE and EMRE results. (b) Estimation of time dependent correlation coefficients using the *Linear Noise Approximation* which are found to be in good agreement with those calculating from ensemble averaging of 5,000 stochastic simulations (c). Note that the oscillatory behavior in the correlation coefficients indicates the presence of noise induced oscillations. The figures were obtained from iNA’s analysis of the SBML file F3.

An enzyme concentration of 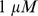 realized in a cellular volume of 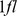 implies a copy number of about 600 enzyme molecules per cell. The *Steady State Analysis* of iNA enables one to obtain a quick overview of the noise characteristics of the reaction network in steady state conditions. Comparing the prediction of the REs and EMREs, as summarized in Fig. 4a, we find excellent agreement at this length scale. Since the average concentrations are well captured by the macroscopic REs we can investigate the fluctuations around them using the LNA. The size of the error bars in Fig. 4a show that as for the Michaelis-Menten reaction, the largest fluctuations in steady-state conditions occur for the substrate species. The mean substrate concentration as obtained from the *table view* is 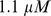 with a CV of about 0.14. This implies fluctuations of roughly 90 substrate molecules showing that molecular fluctuations in enzyme kinetics can be very significant even at the cellular scale.

**Figure 5:**
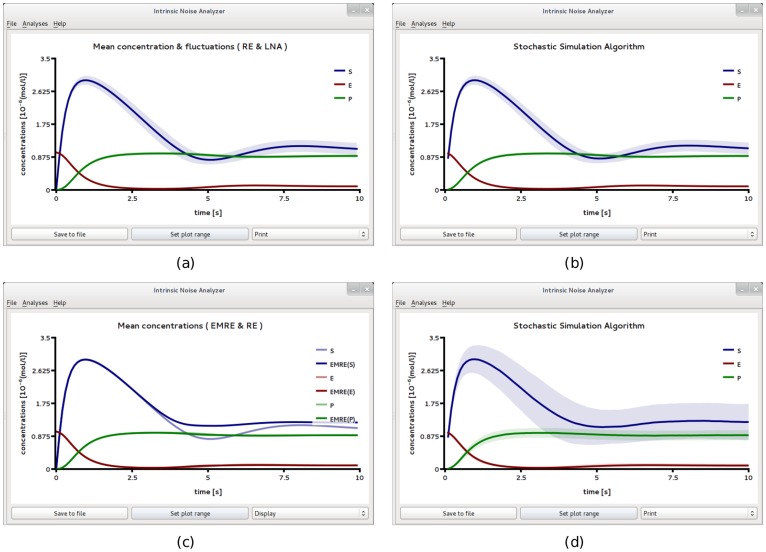
Transient dynamics of cooperative enzyme kinetics in large and small compartments. (a) shows damped oscillations in the mean concentrations of the RE and the fluctuations about them at the cellular scale 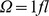 (large compartment) which are in excellent agreement with the ensemble averaged data obtained from 5,000 stochastic simulations (b). In (c) we show how the EMRE predicts that noise destroys the oscillations when the reaction is taking place inside a small intracellular compartment of size 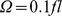. This is well reproduced by stochastic simulations using 10,000 realizations (d). Figure (a) and (b) have been obtained from iNA’s analysis of SBML file F3 while (c) and (d) were obtained from analysis of SBML file F4.

We now use iNA to calculate the transient correlations in the dynamics. Note that the presence of correlations is a distinct feature of the stochastic description. The correlation coefficient as defined by Eq. (8) can be computed by iNA using the LNA, as well as from stochastic simulations, see Fig. 4 b,c. The two are in excellent agreement. The fluctuations of free enzyme and substrate concentrations become anti-correlated as the steady state is approached; this is expected and simply due to the fact that they bind to each other. However, what is more interesting is that the transient correlations can exhibit a complex biphasic behavior, alternating between positively and negatively correlated states as a function of time. Damped oscillations in the correlation coefficients can be traced to the presence of a pair of complex conjugate eigenvalues of the Jacobian of the REs. The latter have been connected with the presence of noise-induced oscillations that are observed in single realizations of the stochastic dynamics [48]. Similar oscillations have also been found for enzymes with negative cooperativity, i.e., in the presence of an inhibitor, and have been related to the presence of damped oscillations in the corresponding deterministic model [49]. Indeed the *Time Course Analysis* carried out by iNA shows transient damped oscillations in the mean concentrations, see Fig. 5a. We have also verified this transient dependence against stochastic simulations which are shown in Fig. 5b and found to be in good agreement with the results of the LNA.

#### Cooperative enzyme kinetics in a small intracellular compartment

Many enzymes operate in small compartments. Hence for physiological concentrations, the enzyme copy numbers can be quite small. In the second SBML model file, F4, we have reduced the compartmental volume to 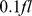 which at constant total enzyme concentration implies a copy number of only 60 enzyme molecules. The time course of the mean concentrations can easily be investigated by means of the EMRE implemented by iNA. The result is shown in Fig. 5c. We observe that the time course is in good agreement with the macroscopic REs only for free enzyme and product concentrations. In contrast, we find that the EMRE predicts the damped oscillations in the substrate concentration to die out quicker than predicted by the REs. This inherent dephasing of individual realizations (compared to the previous case in which the volume was 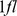) can be traced back to increased noise due to smaller copy numbers at the subcellular scale. We have qualitatively verified this effect by ensemble averaging 10,000 stochastic simulation realizations, see Fig. 5d.

### Gene Regulatory Network with a Negative Feedback Loop

Many genes are represented by only a single copy inside living cells. It is also a fact that many important regulatory molecules exist in low copy numbers. Hence the biochemical process of gene expression is inherently stochastic which also implies that there is a significant amount of noise in the protein levels.

A remarkable property of gene expression is the emergence of cellular rhythms which are commonly attributed to a negative feedback loop in which clock proteins inhibit the expression of their own gene [50]–[52]. There is an ongoing debate whether circadian rhythms (a class of cellular rhythms) observed in constant darkness are self-sustained or noise-induced [53]. The latter, which can be analyzed by means of the SSE, is considered here.

We consider the case where cell-cell coupling and cell-to-cell variability are negligible. Then a single SSA realization of the stochastic dynamics of a circadian clock circuit models single cell circadian rhythms of clock expression while the average over independent realizations models the ensemble rhythm at the population level.

The genetic network under study is a variant of the core model for a circadian clock which has been considered by Gonze and Goldbeter in Ref. [40]. At the heart of the model is a set of effective reactions that describe transcription and translation
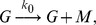


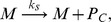


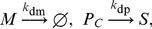
(13)which is a common to many simple models of gene expression [54], [55]. The above involves the clock gene, *G*, the transcribed mRNA, *M* and the cytosolic clock protein, *P_c_*. Note that we have also taken into account mRNA degradation with rate *k_dm_* as well as the consumption of protein with rate *k_dp_* in a different pathway involving *S* which is not explicitly considered here. The negative feedback loop arises from transport of the cytosolic clock protein into the nucleus (the nuclear protein is represented by *P_N_*) [56]

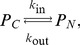
(14)where it binds to promoter regions of DNA while inhibiting the expression of its own gene



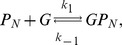


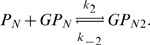
(15)Furthermore we assume that degradation occurs via the following enzymatic processes



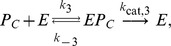


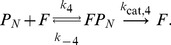
(16)


The constraint that the gene copy number is fixed to one implies that the RE model for this system depends on the compartment volume. Taking into account discreteness, e.g., calculating the EMRE corrections, generates a volume dependence on top of this pre-existing volume dependence. To clearly distinguish the volume corrections due to the EMRE, we follow [35] (see its Supplementary Information) and scale the rates such that we eliminate the volume dependence of the REs. This ensures that the effective rates for transcription and DNA binding are the same for different reaction volumes. The rescaling is as follows: 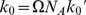, 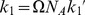, 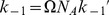, 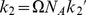 and 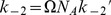. We further impose cooperative binding by the choice of rate constants 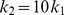 and 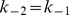. The specific values of the rate constants used in this example can be found in Table 5.

**Table 5:** Rate constants for circadian clock model (SBML files F5 and F6. File F7 has the same constants except that *k*_1_ is multiplied by a factor of 100.).

*k*_in_	5*d* ^−1^	*k*_out_	5*d* ^−1^
*k*_0_′	500*d* ^−1^	*k*_0_	Ω*N_A_k*_0_′
*k*_1_′	0.5*d* ^−1^	*k*_1_	Ω*N_A_k*_1_′
*k*_−1_′	0.5*d* ^−1^	*k*_−1_	Ω*N_A_k_−_*_1_
*k*_2_	10*k*_1_	*k*_−2_	*k*_−1_
*k*_3_	0.5(*µMd*)^−1^	*k*_−3_	0.5*d* ^−1^
*k*_4_	10(*µMd*)^−1^	*k*_−4_	5*d* ^−1^
*k*_cat,3_	0.5*d* ^−1^	*k*_cat,4_	10*d* ^−1^
*k_s_*	5*d* ^−1^	*k_dm_*,*k_dp_*	5*d* ^−1^

Note that the time unit is days (d).

**Figure 6:**
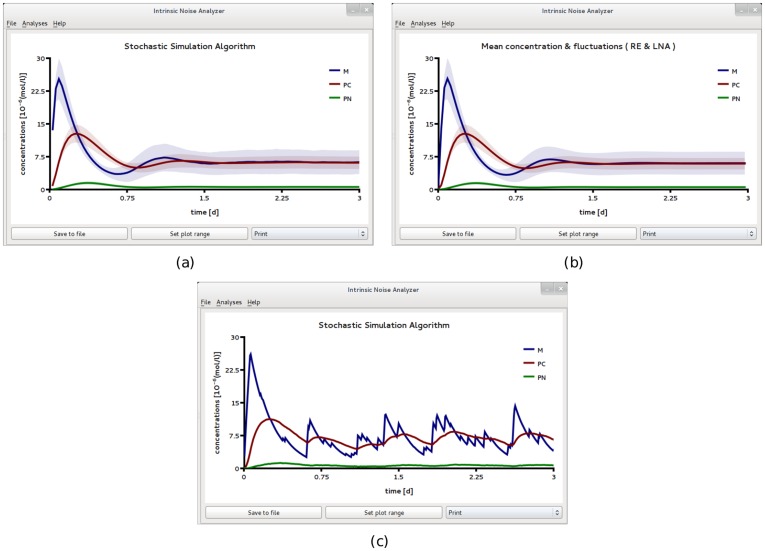
A circadian clock model realized in a large compartment of volume 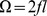. (a) Mean concentrations and variance of fluctuations obtained by ensemble averaging 3,000 stochastic realizations. This corresponds to averages calculated over a population of an equal number of uncoupled identical cells, each having a circadian clock inside. The latter time course is well reproduced by the *Linear Noise Approximation* shown in (b). While the population level concentrations display damped oscillations, individual stochastic simulation realizations (which correspond to individual cell data) reveal no obvious periodicities, (c). All figures have been obtained by analyzing SBML file F5 with iNA.

**Figure 7:**
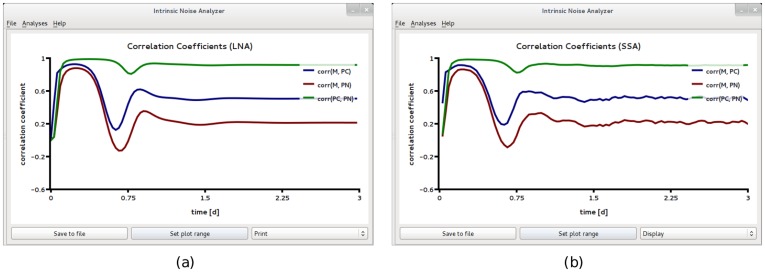
Transients in the correlations of concentration fluctuations for a circadian clock model realized in a large compartment of volume 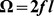. Comparison of the time course of correlations obtained from the *Linear Noise Approximation* (a) and from ensemble averaging stochastic simulations of 3,000 independent cells (b). The two are in good agreement. The minima are a signature of repression due to the negative feedback loop. Both figures have been obtained by analyzing SBML file F5 with iNA.

Two SBML files F5 and F6 were created to describe the biochemical reactions in compartments of size 2*fl* and 0.2*fl* respectively. Figures Fig. 6 and 7 show the results obtained by analyzing file F5 using iNA. At this length scale, the population level descriptions of the mean concentrations and of the variances of fluctuations as given by the REs and the LNA respectively, match very well those obtained from stochastic simulations (compare Fig. 6a and b). In Fig 6c we show a single SSA trajectory which corresponds to single cell level data. Note that while at the population level, one can observe synchronous damped oscillations, at the single cell level oscillations are not evident. In Fig. 7 we show the results of a more detailed test on the accuracy of the LNA. We compare the correlation coefficients estimated from the LNA and stochastic simulations and find good agreement between the two. We observe that the fluctuations of the cytosolic and nuclear protein species are strongly correlated with a clear hierarchy of the correlation coefficients over the whole time course, i.e., 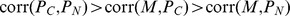. Moreover, we see that over a short time interval *M* and *P_N_* are anti-correlated due to the onset of the repression. The minima in the correlations at some point in time suggests that repression generally affects the fluctuations of all three species.

**Figure 8:**
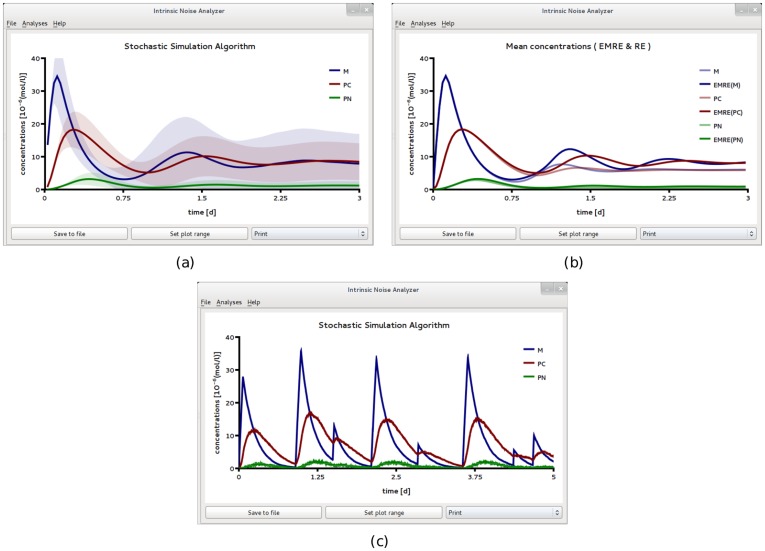
A circadian clock model realized in a small compartment of volume 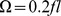 with weak negative feedback. Time course analysis obtained from stochastic simulations of an ensemble of 50,000 independent cells (a) and from the REs and EMREs (b). The EMRE analysis shows synchronous damped oscillations which are amplified compared to the macroscopic REs. This suggests that noise increases the coherence at the single cell level compared to the case 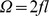, see Fig. 6. This is also supported by single cell stochastic simulations which show bursty noise-induced oscillations of mRNA, *M*, and more regular oscillations for the cytosolic and nuclear protein concentrations, *P_c_* and *P_N_*, respectively. All figures have been obtained by analyzing SBML file F6 with iNA.

It is commonly believed that on average transient responses observed on the cell population level can be accurately described by deterministic REs [57]. This is contrary to the theory of EMREs which predicts that for nonlinear reactions considered in mesoscopic volumes, the mean concentration prediction of the macroscopic REs does not agree with that of the CME. In other words, if the circuit inside each cell is characterized by low copy numbers of interacting molecules then the mean concentrations at the population level (obtained using the SSA) will show deviations from the predictions of the REs. These deviations are too small to observe in Fig. 6. Hence we used iNA to analyze the SBML model definition F6 where the reaction volume is 10 times smaller than that in F5. Figs. 8a and b show the population level *Time Course Analysis* provided by iNA, according to the SSA, EMREs and the REs. Note that the EMRE predictions no longer agree with those of the macroscopic REs. In fact, we find that on the cell population level, the influence of intrinsic noise reduces the damping of synchronous oscillations compared to the large copy number case considered in Fig. 6b. A comparison of Figs. 8a and b shows that these predictions are supported by stochastic simulations. Reduced damping of the oscillations can be interpreted as increased coherence of individual stochastic realizations on the single cell level. We have also carried out single cell simulations, see Fig. 8c, which qualitatively support this conclusion. In fact, we find that the protein concentrations in a single cell exhibit sustained oscillations at a period of about one day which is in agreement with the period of synchronous damped oscillations at the population level, see Fig. 8. Note also the shift to lower frequency in the damped oscillations. Similar noise-induced shifts have been observed by other authors [58] using stochastic simulations.

**Figure 9:**
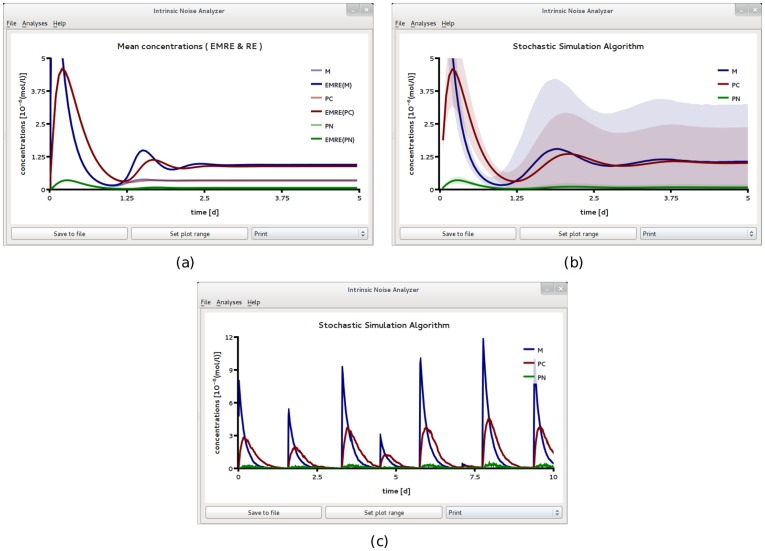
A circadian clock model realized in a small compartment of volume 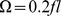 and with strong negative feedback. This rate constant *k*_1_ which controls the strength of transcriptional repression is made a hundred times larger than that used for Fig. 8. In (a) we compare the predictions of the REs with those of the EMREs. The EMREs predict damped oscillations at the population level which are missed by the REs. The presence of these noise-induced synchronous oscillations is qualitatively confirmed by stochastic simulations of 100,000 independent cells (b). However, the phase of the damped oscillation is quantitatively different from the EMREs. In particular, panel (a) shows damped oscillations with a period of about 1 day (d) while the ones obtained from stochastic simulations in panel (b) have a significantly longer period. We have also carried out stochastic simulations at the single cell level (c) which show sustained bursty oscillations with a period of about 1.5 days in the mRNA, *M*, and cytosolic protein, *P_c_*, concentrations. All figures have been obtained by analyzing SBML file F7 with iNA.

To investigate this effect further, we increased the repression rate constant *k*_1_ by a factor of 100, see SBML file F7 in Table 2 with a volume 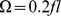. Interestingly, we find that here the EMRE predicts synchronous damped oscillations even when they are absent in the REs, see Fig. 9a. Hence, these oscillations are purely induced by noise since they cannot be observed in the corresponding deterministic model. We have verified this effect against stochastic simulations which are in qualitative agreement as shown in Fig. 9b. Note that although the EMRE prediction is qualitatively correct in predicting oscillations where they are not captured by the REs, the phase and frequency of the oscillations differs significantly from the stochastic simulations at the cell population level, as shown in Fig. 9b. Higher-order corrections to the mean concentrations than those of the EMRE would be needed to correctly capture such details. The theory of such corrections has already been worked out [59] but they are not presently implemented in iNA. While at the cell population level, we have noise-induced damped oscillations, at the single cell level we observe persistent periodic rhythms in the mRNA and protein concentrations (see Fig. 9c) which are considerably more regular and conspicuous compared to the case of weak negative feedback in Fig. 8. Similar differences between the population level and cell level circadian dynamics have been experimentally observed [60].

We conclude by noting that the fact that the RE model could not capture the population level dynamics suggests that the common practice of extracting rate constants by fitting population level data to RE model predictions (see for example [61]) may lead to erroneous results whenever the single cell dynamics are considerably affected by intrinsic noise.

### Design and Implementation

**Figure 10:**
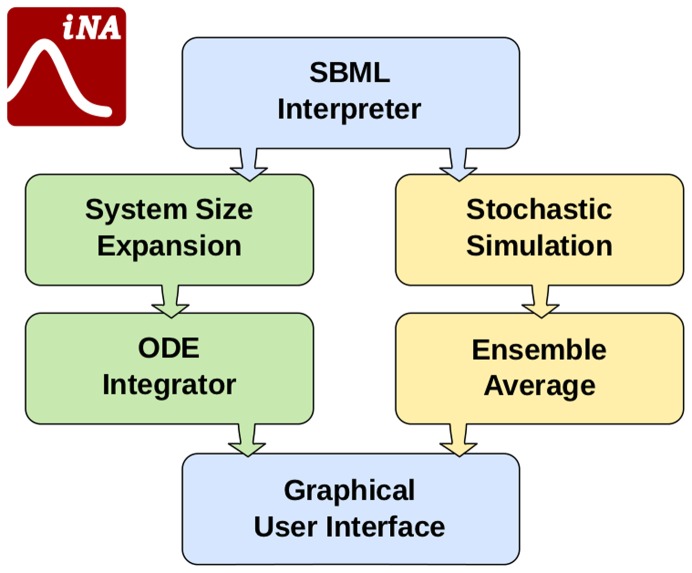
Schematic illustrating the architecture of the software iNA. The software reads an SBML file, sets up an internal mathematical representation of the biochemical reaction scheme, and computes the mean concentrations and the variances and covariance of concentration fluctuations as a function of time by stochastic simulations and by approximations based on the system size expansion. The results are then output via tables and plots implemented in the GUI.

The conceptual design of iNA consists of three layers: model interpretation, computation and GUI which are shown in Fig. 10. The input is a SBML model definition file from which an interpreter of the mathematical model representation is set up. The computational layer of iNA can be accessed by the user in two ways. One route is direct stochastic simulation of the model. This procedure has to be carried out repeatedly in order to obtain sufficiently accurate statistical averages. The second route is to obtain an approximate analytical solution for the mean concentrations and the variance and covariance of concentration fluctuations of the CME. For the latter we employ the Linear Noise Approximation and the EMRE method which rely on van Kampen’s SSE. The output of the computation is visualized by table and plot views implemented in the GUI. iNA has been implemented in C++ and makes use of the cross-platform library Qt (Nokia, Inc) which yields comparable graphical interfaces on different platforms from a single source code.

#### The SBML parser

The model file is parsed using the library libSBML [62] from which the interpreter is set up. The latter constructs a mathematical representation of the model which is suitable for the use of the computer algebra system Ginac [63]. Such representation comprises the list of species, the stoichiometric matrix and the set of propensities characterizing the reaction network. Though symbolic representations of SBML are available [64], these libraries are limited to the representations of models using deterministic REs. The implementation presented here is the first to adopt this approach for the computation of the SSE of the CME, which goes beyond the validity of the deterministic REs. Thereby it fills the gap between analytical approximations and stochastic simulation of biochemical kinetics.

#### SSA and methods based on the SSE

The computational layer offers two methods for the stochastic simulation: the direct method developed by Gillespie [25] and the more recently introduced optimized direct method [27]. It is our experience that the latter typically speeds up simulations by a factor of two. The computation of the LNA and EMRE requires the series expansion of the CME in powers of the inverse square root of the volume of the compartment (see the section *Methods*). We make use of the library Ginac, by means of which we can compute derivatives analytically and obtain series expansions of the mathematical model representation instead of resorting to numerical approximations. The computations are heavily based on basic linear algebra, for which we resort to the C++ template library Eigen [65] that has been customized to work with symbolic representations of Ginac. In order to allow for unconstrained numerical integration, it is also necessary to identify the corresponding conservation laws of the reaction network which is done using the full-pivoting LU decomposition provided by Eigen. This algorithm has been described in Ref. [66] and has been shown to give reliable results. The numerical integration of the ordinary differential equations describing REs, the LNA and EMREs is performed using explicit Runge-Kutta algorithms (RK4, RKF45, Dopri54) or implicit Rosenbrock method (4/5th order) for stiff problems [36], [67], [68].

#### Optimizations

Generally the number of simultaneous equations that need to be solved by the software is given by N for the REs, 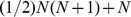 for the LNA and 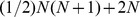 for the EMREs, where N is the number of species (see the section *Methods*). Hence the complexity of the SSE based methods grows at least quadratically with the number of species depending on the algorithm of integration employed. In contrast, the performance of the SSA is limited first by the number of reactions over the time course, which is proportional to the size of the propensity and second by the large number of realizations required to obtain accurate statistical averages. We have addressed the latter issue by supplying our stochastic simulators with easy-to-use OpenMP parallelism which is available on many platforms and can be accessed through the *Stochastic Simulation* wizard. The remaining bottlenecks concern the performance of expression evaluation which is at the heart of both analytical and stochastic simulation methods. In order to allow for efficient evaluation of the required expressions, we have set up a bytecode interpreter, a concept which is common to dynamic programming languages. Bytecode interpreters increase the execution performance by reducing the code complexity and allow for very efficient implementations while at the same time maintaining the portability of code across many platforms. The expressions computed by iNA are first compiled into a bytecode representation which is then reduced by fast peep-hole optimizations [69] and finally evaluated using an efficient interpreter implementing stack-machines. On single-core architectures we observed a speed up by a factor of 10−20 compared to the use of conventional methods for both stochastic simulations and SSE based methods. On multi-core architectures the performance of the SSE methods could be significantly improved by the use multiple stacks enabling parallel expression evaluation.

## Methods

### General Formulation of the SSE

In the section *The Chemical Master Equation and the System Size Expansion* we have introduced the CME, Eq. (3). The latter is typically intractable for analytical solution. In fact, exact solutions exist only for a handful of cases. However, the dynamics can be investigated systematically by means of van Kampen’s SSE [9], [70]. The starting point of the analysis is the so called Van Kampen ansatz
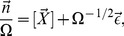
(17)


by which one separates the instantaneous concentration into a deterministic part given by the solution 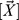 of the macroscopic REs, Eq. (4), and the fluctuations around it. The change of variable causes the probability distribution of molecular populations 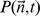 to be transformed into a new probability distribution of fluctuations 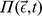. In particular, the time derivative transforms as [9]

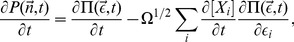
(18)


which takes into account the change in probability along the deterministic trajectory of 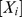.

#### The expansion of the step operator

The core of the expansion now follows from the observation that the step operator can be written as the Taylor series 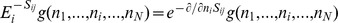 which upon the change of variable, Eq. (17), can be expanded in terms of the inverse square root of the volume 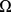.
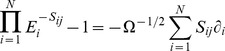


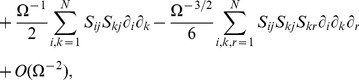
(19)


where 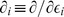.

#### The expansion of the propensity

We now turn to the expansion of the propensity which is done in two steps. Note that from here on we use the convention that twice repeated Greek indices have to be summed over 1 to N. First we expand the propensities in powers of epsilon. The result is
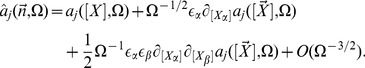
(20)


Note that 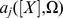 is simply the microscopic propensity evaluated at the deterministic concentrations. As shown below the latter depends explicitly on 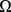 and can be expanded into a finite series of the form
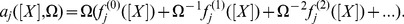
(21)


The first term denoted by 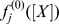 is typically associated with the macroscopic rate function of the *j*-th reaction. Combining Eq. (20) and (21) we find
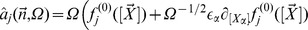


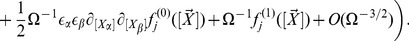
(22)


In this way we obtain an expansion whose coefficients are independent of 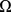. We will illustrate the expansion for the case of mass action kinetics and the case of non-elementary reactions. The former case allows to write the propensity in the generic form
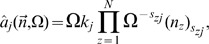
(23)


where 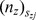 is the falling factorial 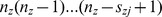. The microscopic propensity becomes
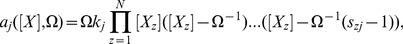
(24)


and hence by collecting terms of order 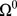 and 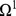 it follows that
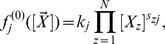


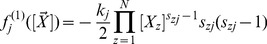
(25)


For non-elementary reactions we consider the Michaelis-Menten example with reaction propensity 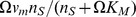 as has been suggested by Rao [71]. By expressing the particle number in terms of concentrations one observes that by construction macroscopic and microscopic propensities agree, i.e., we have 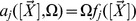. It follows that the macroscopic rate function becomes 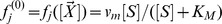 while 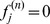 for all *n*>0. The above expansions are computed automatically by iNA and do not require any further user input. Note however that in the situation where the user defined propensity cannot be expanded in the prescribed way the software simply assumes that 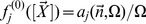 at constant 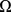. By doing so the correctness of the numerical values used in the analysis is ensured.

#### The expansion of the CME

The expansion obtained in this way can be written as
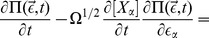


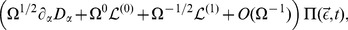
(26)


where the individual terms in the expansion are obtained using (3) together with the expansions (19), (20) and (21) and grouping terms in descending powers of 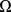.
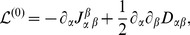
(27)

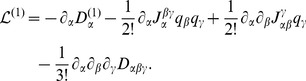
(28)Note that Eq. (28) corrects Eq. 14 in Ref. [31] which is missing the third term. However, this does not affect the result. Here we have the defined the SSE coefficients given by



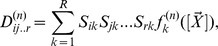


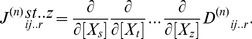
(29)


Note that we adopt the convention to omit the index for *n* = 0, e.g., 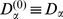. We can now equate the terms of order 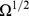 which appear on the left and right hand side of Eq. (26):
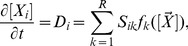
(30)which are the macroscopic REs.

#### The linear noise approximation

We now proceed by constructing equations for the moments of the 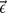 variables. We follow the derivation presented in Ref. [59] and expand the probability distribution of fluctuations 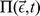 in terms of the inverse square root of the volume
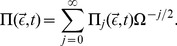
(31)In consequence there exists an equivalent expansion of the moments
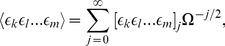
(32)where the following definition has been used



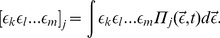
(33)Using the expansion of the probability density, Eq. (31), together with Eq. (26) and (27) we find after equating all terms of order 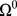:
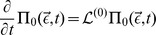


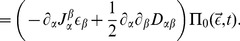
(34)


The result is the Linear Noise Approximation which yields a linear Fokker-Planck equation. Its solution is well known to be a multivariate Gaussian distribution [9], [29]. The time evolution equations of the first two moments are obtained by multiplying the latter by 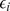 and 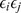 respectively and performing the integration over 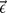:
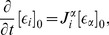
(35)

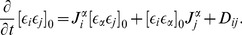
(36)


Note that in the case of the deterministic initial conditions we have 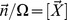 initially and hence all moments of 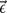 given by Eq. (32) are zero for *t* = 0. We can also deduce by inspection of Eq. (35) that in this case we have 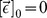 for all times. In order to relate the moments back to the moments of the concentration variables we use Eq. (32) and (17) to find that mean concentrations and covariance matrix are given by
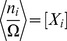
(37)

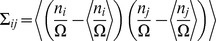


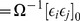
(38)


Using the definition of the covariance matrix 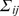 together with Eq. (36) we find that it satisfies the time dependent matrix equation
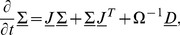
(39)


where the Jacobian and diffusion matrix are given by 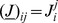 and 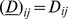, respectively [29]. It follows that to order 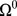 the average concentrations are correctly described by the macroscopic REs. The size of fluctuations about the average are then given by the matrix 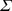 as obtained from the solution of Eq. (39).

#### Effective Mesoscopic Rate Equations

In this section we will use higher order corrections to derive a set of effective mesoscopic rate equations, the EMREs, that are expected to be closer to the true concentrations as predicted by the CME. We therefore utilize the system size expansion, Eq. (27) together with Eq. (31) and equate terms of the order 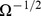. The result is given by
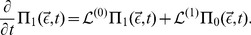
(40)


Multiplying the above equation by 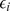 and performing the integration over 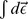 we find
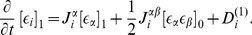
(41)


We can use the above result together with Eq. (17) and (32) to find an equation for the average concentration accurate to order 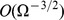:
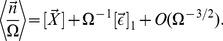
(42)Hence we conclude that the difference between the true concentrations predicted by the CME and those of the macroscopic RES evolves as



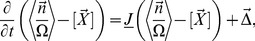
(43)where we have defined the vector 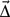 with components
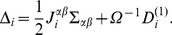
(44)


Note that in the case of deterministic initial conditions this result enjoys an increased accuracy of order 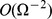 as has been shown in Ref. [59]. Note that by virtue of Eq. (17) we see that each nonzero solution of the above equation implies a correction to the concentrations as macroscopic REs. Necessary conditions for this to be true depend on the explicit form of the propensities such as propensities with nonlinear dependence on the molecular populations for which 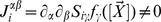 or propensities for which microscopic and macroscopic rate functions do not agree, i.e., 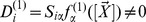. Note that for a network composed entirely of elementary reactions 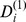 is non-zero only for species which dimerize because of the special form of the propensity which is given by 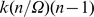. It can be shown that in this case 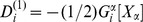 with 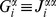 and hence
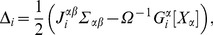
(45)which agrees with the result given by Grima for networks composed of elementary reactions [31].

### Concentration Inversion Effect for the Michaelis-Menten Reaction

Recently, Ramaswamy et al. [35] have reported a noise-induced concentration inversion effect using the EMREs. The authors consider independent realizations of the same chemical system in compartments of different volumes. They find that for monostable reaction networks with bimolecular reactions, when a species is more more abundant than another for large compartmental volumes, i.e., the regime where the REs are valid, then the reverse occurs for an identical system realized in compartmental volumes below a critical value, i.e., the regime where the EMREs give a more accurate description of the true mean concentrations.

We here derive the equation for the critical volume for this discreteness-induced concentration inversion of substrate and product in the Michaelis-Menten scheme (10). The macroscopic REs are given by
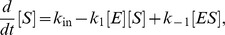


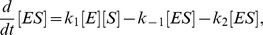


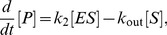
(46)


where 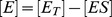 by conservation of total enzyme concentration. Under steady state conditions the above equations can be solved by setting the time derivatives to zero which yields 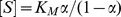, 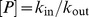 and 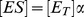 where 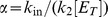 is measuring the fractional enzyme saturation. The EMREs of the reaction have been first obtained by Grima in Ref. [32] where it was found that the REs are only accurate for describing the enzyme and product concentrations in mesoscopic volumes, i.e., we have 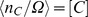 and 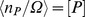. However they underestimate the mesoscopic substrate concentration. The critical volume under which the concentrations of substrate and product concentrations are equal is obtained from the condition
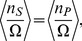
(47)which implies the same equality in terms of molecule numbers. The finite volume correction to the macroscopic REs as given in Ref. [33], Eq. 29, leads to the condition



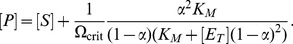
(48)Solving the above equation for 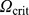 we find
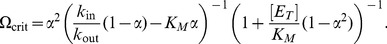
(49)


Using the rate constants for the Malate dehydrogenase enzyme summarized in Table 3 we find a critical volume of 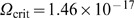.
